# Humanized CXCL12 antibody delays onset and modulates immune response in alopecia areata mice: insights from single-cell RNA sequencing

**DOI:** 10.3389/fimmu.2024.1444777

**Published:** 2024-10-17

**Authors:** Seungchan An, Mei Zheng, In Guk Park, Sang Gyu Park, Minsoo Noh, Jong-Hyuk Sung

**Affiliations:** ^1^ College of Pharmacy, Natural Products Research Institute, Seoul National University, Seoul, Republic of Korea; ^2^ Epi Biotech Co., Ltd., R&D Center, Incheon, Republic of Korea; ^3^ College of Pharmacy, Ajou University, Suwon, Republic of Korea

**Keywords:** CXCL12, humanized antibody, alopecia areata, CD8 + T cell, interferon-gamma

## Abstract

It has been demonstrated that CXCL12 inhibits hair growth via CXCR4, and its neutralizing antibody (Ab) increases hair growth in alopecia areata (AA). However, the molecular mechanisms have not been fully elucidated. In the present study, we further prepared humanized CXCL12 Ab for AA treatment and investigated underlying molecular mechanisms using single-cell RNA sequencing. Subcutaneous injection of humanized CXCL12 Ab significantly delayed AA onset in mice, and dorsal skin was analyzed. T cells and dendritic cells/macrophages were increased in the AA model, but decreased after CXCL12 Ab treatment. Pseudobulk RNA sequencing identified 153 differentially expressed genes that were upregulated in AA model and downregulated after Ab treatment. Gene ontology analysis revealed that immune cell chemotaxis and cellular response to type II interferon were upregulated in AA model but downregulated after Ab treatment. We further identified key immune cell-related genes such as *Ifng*, *Cd8a*, *Ccr5*, *Ccl4*, *Ccl5*, and *Il21r*, which were colocalized with *Cxcr4* in T cells and regulated by CXCL12 Ab treatment. Notably, CD8+ T cells were significantly increased and activated via Jak/Stat pathway in the AA model but inactivated after CXCL12 Ab treatment. Collectively, these results indicate that humanized CXCL12 Ab is promising for AA treatment via immune modulatory effects.

## Introduction

CXCL12 is a CXC chemokine traditionally classified as a homeostatic chemokine, contributing to physiological processes such as embryogenesis, hematopoiesis, and angiogenesis (1). In contrast to these homeostatic functions, increased expression of CXCL12 in general, or of a specific CXCL12 splicing variant, has been demonstrated in various pathologies (2, 3). Administration of CXCL12-neutralizing antibodies (Abs) or small-molecule antagonists of CXCR4 delays disease onset or prevents disease progression in cancer, viral infections, inflammatory bowel diseases, rheumatoid arthritis, and osteoarthritis (4, 5). The CXCL12/CXCR4/ACKR3 axis constitutes a potential therapeutic target for a wide variety of inflammatory diseases, not only by interfering with cell migration but also by modulating immune responses (5). In the skin, CXCL12 is highly expressed in dermal fibroblasts (DFs) and mediates inflammatory diseases. For example, CXCL12^+^ DFs promote neutrophil recruitment and host defense through recognition of IL-17 (6). DFs underpin skin immune responses, and cross-talk among DFs, macrophages, and migratory immune cells, including T cells, dendritic cells (DCs), and natural killer (NK) cells in the skin, is important. These skin-resident cells are attracting interest as therapeutic targets in skin inflammatory diseases (7). As described, DFs secrete inflammatory factors such as CXCL12 and play a key role in skin inflammation. However, its role has not been demonstrated in alopecia areata (AA).

In hair biology, we first found that CXCL12 is secreted in DFs and outer root sheath (ORS) cells, and regulates the hair cycle to induce the hair regression period (8). Conversely, inhibition of CXCL12 and CXCR4 promoted hair growth in animal experiments. In androgenic alopecia (AGA) mice, CXCL12 is up-regulated in DFs, and CXCL12-neutralizing Ab promoted hair growth in a dose-dependent manner. Androgens such as testosterone and dihydrotestosterone upregulate CXCL12 via the androgen receptor, and they are colocalized in DFs (9). Subcutaneous injection of CXCL12 Ab reduced the expression of CD8+ and MHC+ cells, thereby improving hair growth in AA animals and preventing AA onset. However, the underlying molecular mechanisms improving AA in mice have not been fully elucidated.

AA pathogenesis is due to the loss of immune privilege of the hair follicle, leading to autoimmune attack. Lee et al. investigated skin-infiltrating immune cells using single-cell analysis from the graft-induced C3H/HeJ mouse model of AA, and found that only the depletion of CD8+ T cells, but not CD4+ T cells, NK cells, B cells, or γδ T cells was sufficient to prevent and reverse AA (10). Although the literature has focused on CD8+ T cells, vital roles for CD4+ T cells and antigen-presenting cells have also been suggested (11). Of interest, one of the CXCL12 receptors, CXCR4, is highly expressed in T cells such as CD8+ T cells and mediates the migration of T cells (12). Conversely, the CXCR4 antagonist AMD3100 inhibited skin inflammation associated with reduced inflammatory cell accumulation (13). As described, AA is an autoimmune disease primarily mediated by skin-resident T cells, and CXCL12-neutralizing Ab is promising for AA treatment. Consequently, a novel humanized Ab for CXCL12 has been developed for non-clinical study, and its underlying molecular mechanism for AA treatment has been elucidated using single-cell RNA sequencing in the present study.

## Methods

### Preparation of single-cell suspension

Skin samples were collected from unaffected C3H/HeN mice (negative control, Neg), AA model mice, and AA model mice treated with CXCL12-neutralizing antibody (AA + Ab). Skin from the back of 7-8 mice per group was pooled, minced, and digested with 0.7 mg/mL collagenase D solution (Sigma-Aldrich), and then passed through 70 and 40 μm meshes. Red blood cells were lysed using ACK Lysing Buffer (Gibco), and debris were removed through density gradient-based separation using Percoll (Sigma-Aldrich) media. The cell suspension was immediately processed for single-cell RNA sequencing (scRNA-seq).

### Droplet-based scRNA-seq

Prepared single-cell suspensions were processed using the Chromium Next GEM Single Cell 3’ RNA library v3.1 protocol (10x Genomics) according to the manufacturer’s instructions. Briefly, cells were encapsulated into nanoliter-scale Gel Beads-in-emulsion (GEMs) containing barcoded oligonucleotides. The poly(dT) primers in the GEMs captured polyadenylated mRNA from each cell, allowing for the generation of barcoded, full-length cDNA. The cDNA was amplified to construct 3’ gene expression libraries, which were sequenced on an Illumina sequencing system (Illumina) at Macrogen. Raw BCL files produced by the Illumina platform were demultiplexed into FASTQ files using Cell Ranger v7.2.0 (10x Genomics). Then, *cellranger count* pipeline was used to align the reads onto the mouse reference genome ‘mm10’, and to perform filtering, barcode counting, and unique molecular identifier (UMI) counting. The sequencing data quality was assessed using FastQC v0.11.7.

### Single-cell transcriptome analysis

The analysis targeted a total of 28,351 skin cells across three groups (Neg, AA, and AA + Ab). Initial processing was conducted using the R package Seurat v5.0.3 including cell filtering, clustering, annotation and visualization, as previously described (14, 15). Cells with over 10% mitochondrial gene expression or expressing fewer than 200 genes were excluded. Putative multiplet cells were also removed using DoubletFinder v2.0.4 with default parameters (16). After this quality control process, 23,222 cells from the three samples were retained for analysis. The count matrix was then normalized, and 2,000 highly variable genes were selected for scaling. Cell clustering was performed using a shared nearest-neighbor method, followed by the Louvain algorithm for modularity optimization and clustering. The subsequent dimensionality reduction was achieved by t-distributed stochastic neighbor embedding (t-SNE) or uniform manifold approximation and projection (UMAP). Clusters were manually annotated based on their expressing genes using literature-based markers (17–19). To validate our annotation strategy, we visualized the expression of canonical or published marker genes for each cell type. Additional subpopulation identification for T cells, fibroblasts, monocytes, dendritic cells, and macrophages was performed following the above procedures. Pseudotime trajectory analysis was performed for naïve-like and CD8+ T cells using monocle3 v1.3.7 (20). Cell-cell communication was profiled using CellChat v2.1.1 (21).

### Differential expression analysis

Initial differential expression analysis among the Neg, AA, and AA + Ab groups was conducted after aggregating gene expression counts from all cells using *PseudobulkExpression* module of Seurat package. Genes with a twofold expression difference between groups were identified as differentially expressed genes (DEGs). These DEGs were further analyzed for protein-protein interaction networks and functional enrichment using the STRING server (22, 23), gprofiler2 v0.2.3 (24) or GSEA v4.0.3 (25). Community detection in STRING network of DEGs was performed using R package igraph v2.0.3. For the cell-type level identification of DEGs, the *FindMarkers* module of Seurat package was used.

## Results

### scRNA-seq analysis revealed biased cell populations in AA

**Figure 1:**
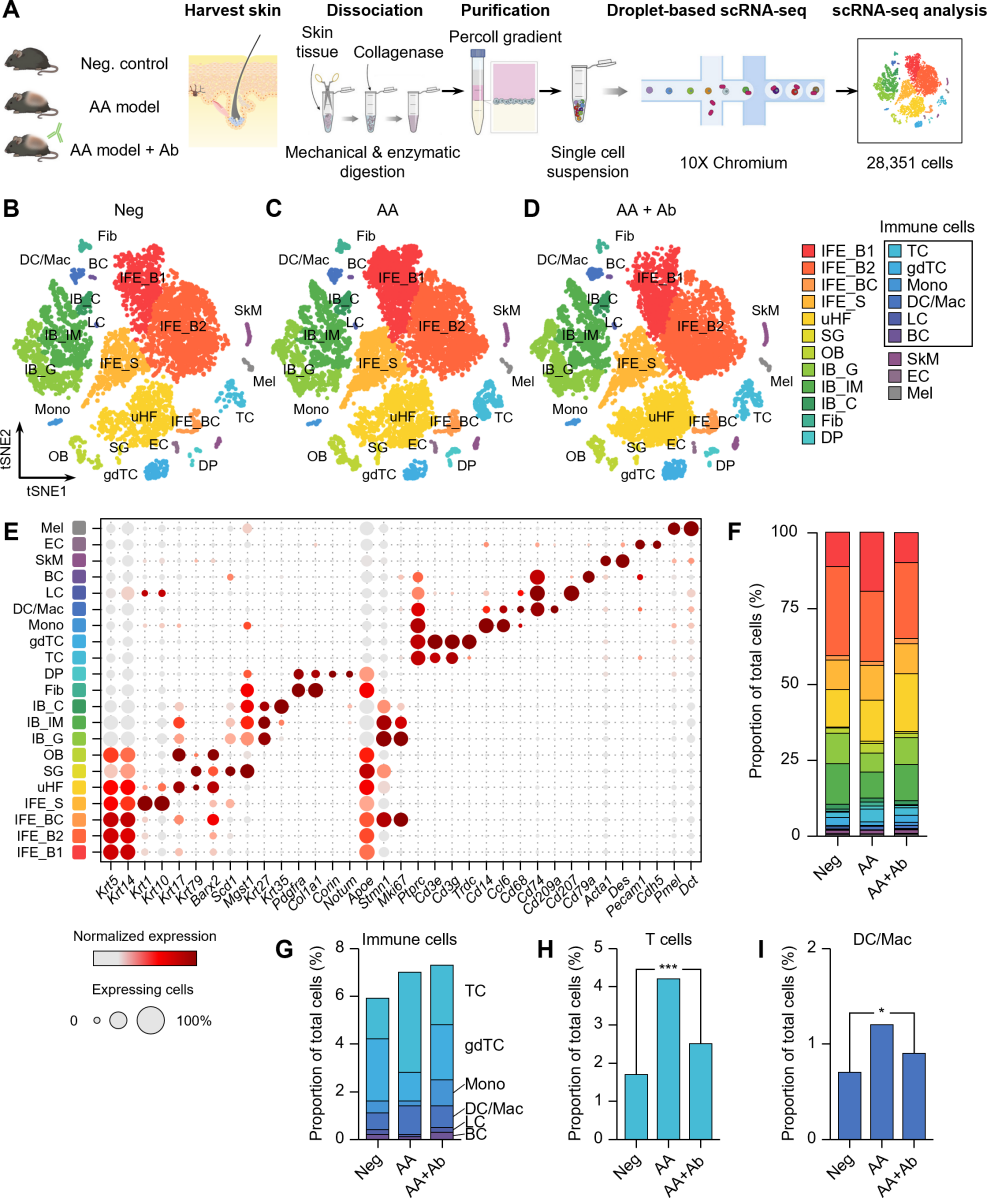
scRNA-seq of CXCL12 Ab-treated AA mouse model. **(A)** Workflow of scRNA-seq analysis. **(B–D)** t-SNE representation of scRNA-seq results from **(B)** Neg, **(C)** AA, and **(D)** AA + Ab groups, colored by annotated cell types. **(E)** Expression levels for selected marker genes of each cell type. **(F)** Proportion of each cell type among total cells for each group. **(G–I)** Proportion of **(G)** immune cells, **(H)** T cells, and **(I)** DC/Mac among total cells for each group. Statistical significance was assessed using the binomial test. *P < 0.05, ***P < 0.001.

Skin samples were collected from unaffected mice (negative control, Neg), AA model mice, and AA model mice treated with CXCL12-neutralizing antibody (AA + Ab) (**Supplementary Figure S1**). Following skin tissue dissociation, purification and droplet-based scRNA-seq, we obtained transcriptomic profiles of 28,351 cells, where 23,222 cells passed primary quality control filter (**Figure 1A**; **Supplementary Figure S2**). Based on cell-specific markers, we identified at least 15 distinct cell types across all three groups, representing the full thickness of the skin (**Figures 1B–D**). The predominant cell population was interfollicular epidermis (IFE) keratinocytes, characterized by high expression of *Krt5* and *Krt14* (**Figure 1E**). Within this population, we distinguished basal IFE keratinocytes (IFE_B), proliferating IFE keratinocytes (marked by elevated *Stmn1* and *Mki67* expression; IFE_BC), and spinous IFE (IFE_S) keratinocytes (expressing *Krt1* and *Krt10* expression). These IFE cells comprised 47-55% of the total cell population (**Figure 1F**). Additional cell types identified included upper hair follicle (uHF) keratinocytes, outer bulge (OB) keratinocytes, and sebaceous gland (SG) cells, marked by the expression of *Krt79*, *Barx2*, and *Mgst1*, respectively (**Supplementary Table S1**). Inner bulge (IB) keratinocytes were characterized by *Krt27* and *Krt35* expression, with IB cells of germinative layer (IB_G) showing high *Stmn1* and *Mki67* expression (**Figure 1E**).

Beyond keratinocytes, the scRNA-seq analysis also yielded a diverse array of fibroblasts, immune cells, skeletal muscle cells, and melanocytes (**Figure 1E**; **Supplementary Table S1**). We identified fibroblasts highly expressing *Pdgfra* and *Col1a1*, and dermal papilla (DP) cells expressing *Corin* and *Notum*. Endothelial cells (EC) were characterized by *Pecam1* and *Cdh5* expression. Among CD45+ (*Ptprc* gene) immune cells, we identified T cells (TC; expressing *Cd3e*), γδT cells (gdTC; *Trdc*), monocytes (Mono; *Cd14* and *Ccl6*), dendritic cells and macrophages (DC/Mac; *Cd68*, *Cd74*, and *Cd209a*), Langerhans cells (LC; *Cd207*), and B cells (BC; *Cd79a*) (**Figure 1E**). Notably, the proportion of immune cells increased in the AA model, with significant rises in the TC and DC/Mac populations, which decreased following CXCL12 Ab treatment (**Supplementary Figure S3**). The proportion of T cells across the groups was 1.7%, 4.2%, and 2.5% for Neg, AA, and AA + Ab, respectively, while the DC/Mac proportions were 0.7%, 1.2%, and 0.9% (**Figures 1H, I**).

### Pseudobulk RNA-seq analysis revealed altered immune response in AA

**Figure 2:**
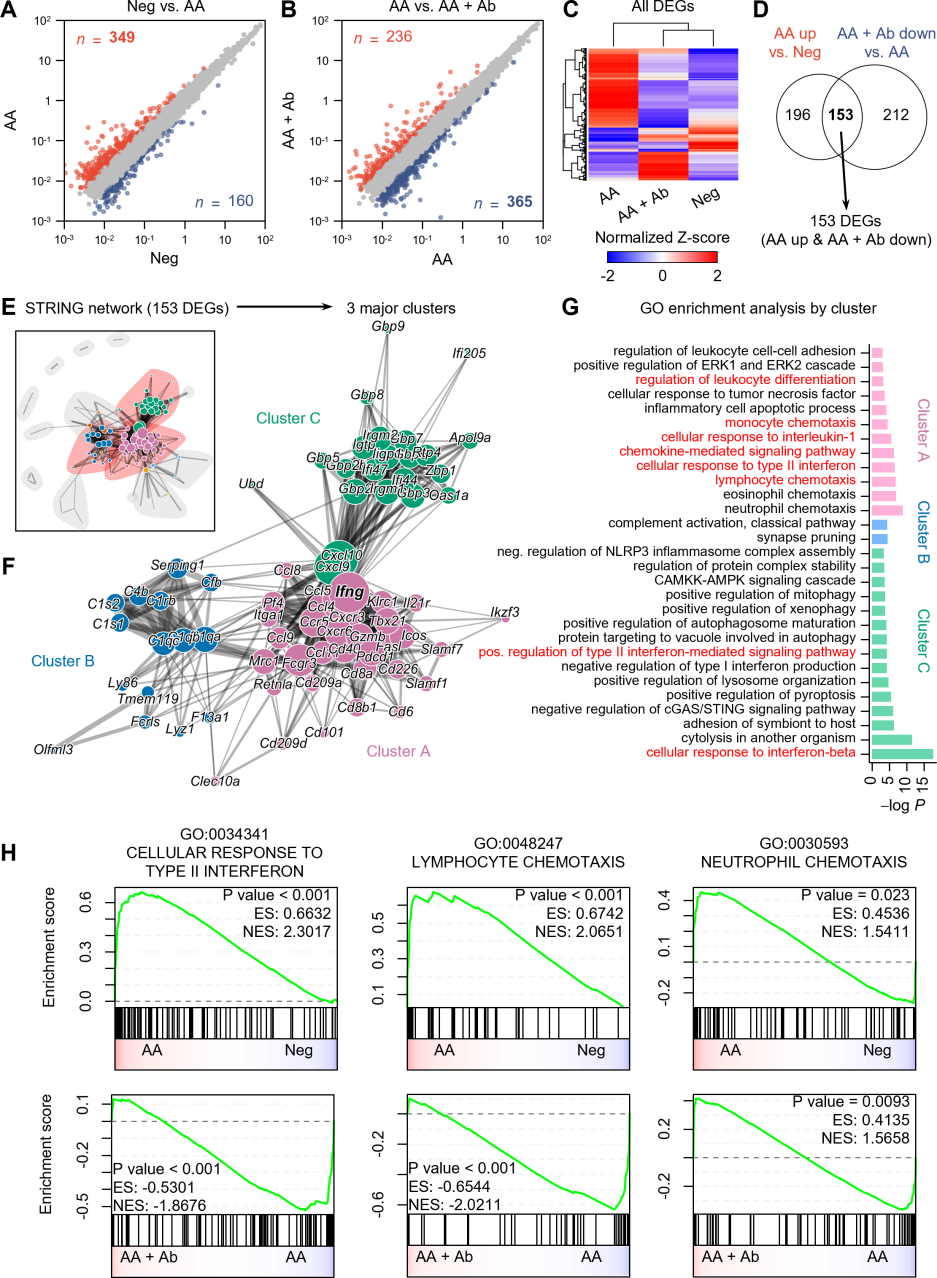
Pseudobulk RNA-seq analysis of CXCL12 Ab-treated AA mouse model. **(A, B)** Identification of DEGs: **(A)** Neg vs. AA and **(B)** AA vs. AA + Ab. Genes with a more than twofold increase are shown in red, and those with a decrease are shown in blue, with the number of each DEG indicated. **(C)** Heatmap showing the normalized Z-scores of all DEGs. **(D)** Venn diagram depicting the identification of 153 DEGs (AA up & AA + Ab down). **(E)** STRING network based on protein-protein interactions of the 153 DEGs. **(F)** Three major clusters are identified from community detection based on weighted edge betweenness. **(G)** Gene ontology (GO) enrichment analysis results for each cluster. Significant results with *P* value < 0.001 are shown. **(H)** Pre-ranked GSEA results using log_2_ fold change from each comparison as input.

scRNA-seq analysis indicated significant shifts in immune cell proportions in the AA model. To elucidate the transcriptional changes underlying these shifts, we next performed differential expression analysis and functional enrichment analysis on pseudobulk RNA-seq data aggregated from transcript counts of all cells for each group (**Figure 2**). Compared to the normal (Neg) group, 349 genes showed more than twofold increased expression, and 160 genes showed decreased expression in the AA model (**Figure 2A**). Compared to the AA model, the AA + Ab group had 236 genes with increased expression and 365 genes with decreased expression (**Figure 2B**). Analysis of the expression patterns of all DEGs indicated that approximately 78% of DEGs that were upregulated in the AA model compared to the Neg group subsequently decreased following antibody treatment, and vice versa. This pattern suggests that antibody treatment may help normalize the transcriptional alterations typical of the AA model (**Figure 2C**). We specifically analyzed 153 DEGs that increased in the AA model and decreased following Ab treatment, which likely represent key mediators of both AA pathogenesis and its amelioration through CXCL12 Ab intervention (**Figure 2D**; **Supplementary Figure S4**).

STRING network analysis grouped these 153 DEGs based on their protein-protein interactions (**Figure 2E**), from which we identified three major clusters, A, B, and C, which included 62.5% of the DEGs (**Figure 2F**). Enrichment analysis of each DEG cluster showed that cluster A was significantly associated with pathways related to immune cell chemotaxis (including lymphocytes and monocytes), chemokine-mediated signaling, cellular response to type II interferon, and regulation of leukocyte differentiation (**Figure 2G**). Cluster C was enriched for cytokine response pathways, including responses to type I and II interferons. Meanwhile, genes in cluster B were predominantly linked to the complement system related to functions of dendritic cells and macrophages (26). In addition to the 153 common DEGs, we conducted an analysis of DEGs specifically modulated by the CXCL12 antibody. When applying the same analytical approach as for the common DEGs, we found that the antibody induced relatively few significant changes in biological processes, suggesting minimal off-target effects unrelated to AA treatment (**Supplementary Figure S5**). While the common DEGs were significantly associated with at least 30 biological processes, the antibody-specific DEGs (upregulated and downregulated) were linked to only 5 and 7 biological processes, respectively, despite the similar or higher number of DEGs compared to the common set (**Figure 2G**; **Supplementary Figure S5**). This indicates that the CXCL12 antibody demonstrates a high degree of safety with minimal unintended effects.

Specifically, we observed that the CXCL12 antibody increased the expression of genes involved in the TLR receptor pathway, part of the chemokine signaling network (**Supplementary Figure S5A**). This suggests potential immunomodulatory effects, possibly enhancing innate immune responses. The activation of this pathway may reflect either a specific off-target effect or a therapeutic mechanism relevant to AA. TLR signaling could contribute to AA progression by supporting protective immune responses, but further research is required to clarify these effects. Additionally, the antibody reduced the expression of genes related to calcium transport, muscle contraction, and cell-matrix adhesion (**Supplementary Figure S5B**). Although these reductions may suggest off-target effects impacting calcium signaling and muscle function, the relatively modest nature of these changes implies limited relevance to AA pathogenesis or treatment. Further investigation would be necessary to assess their broader clinical implications. Gene Set Enrichment Analysis (GSEA) conducted on the entire transcriptome also highlighted increased activity of pathways such as cellular response to type II interferon (GO:0034341) and lymphocyte chemotaxis (GO:0048247) in the AA model compared to the Neg group, both of which significantly decreased following Ab treatment (**Figure 2H**; **Supplementary Tables S2**, **S3**). Additionally, pathways related to the cellular response to interferon-beta and regulation of T cell activation were significantly enriched in the AA model compared to the Neg group. Although the regulation of T cell activation pathway was not significantly suppressed following Ab treatment, a trend towards suppression was observed (**Supplementary Figure S5C**).

Genes involved in the cellular response to type II interferon, such as *Ccl5* (C-C motif chemokine ligand 5), *Cd40* (CD40 antigen), *Fasl* (Fas ligand), *Mrc1* (mannose receptor, C type 1), *Ifng* (interferon gamma), and genes encoding guanylate binding proteins (*Gbp2*, *Gbp5*, *Gbp6*, *Gbp8*, and *Gbp9*) were notably upregulated in the AA model compared to Neg and subsequently decreased more than twofold with Ab treatment (**Supplementary Tables S2**). Representative genes associated with chemotaxis included *Ccl5*, *Cxcr3* (C-X-C motif chemokine receptor 3), and *Tnfsf14* (tumor necrosis factor ligand superfamily, member 14) (**Supplementary Tables S3**).

### Cell-cell communication analysis revealed CXCR4 involvement in immune cell activation

**Figure 3:**
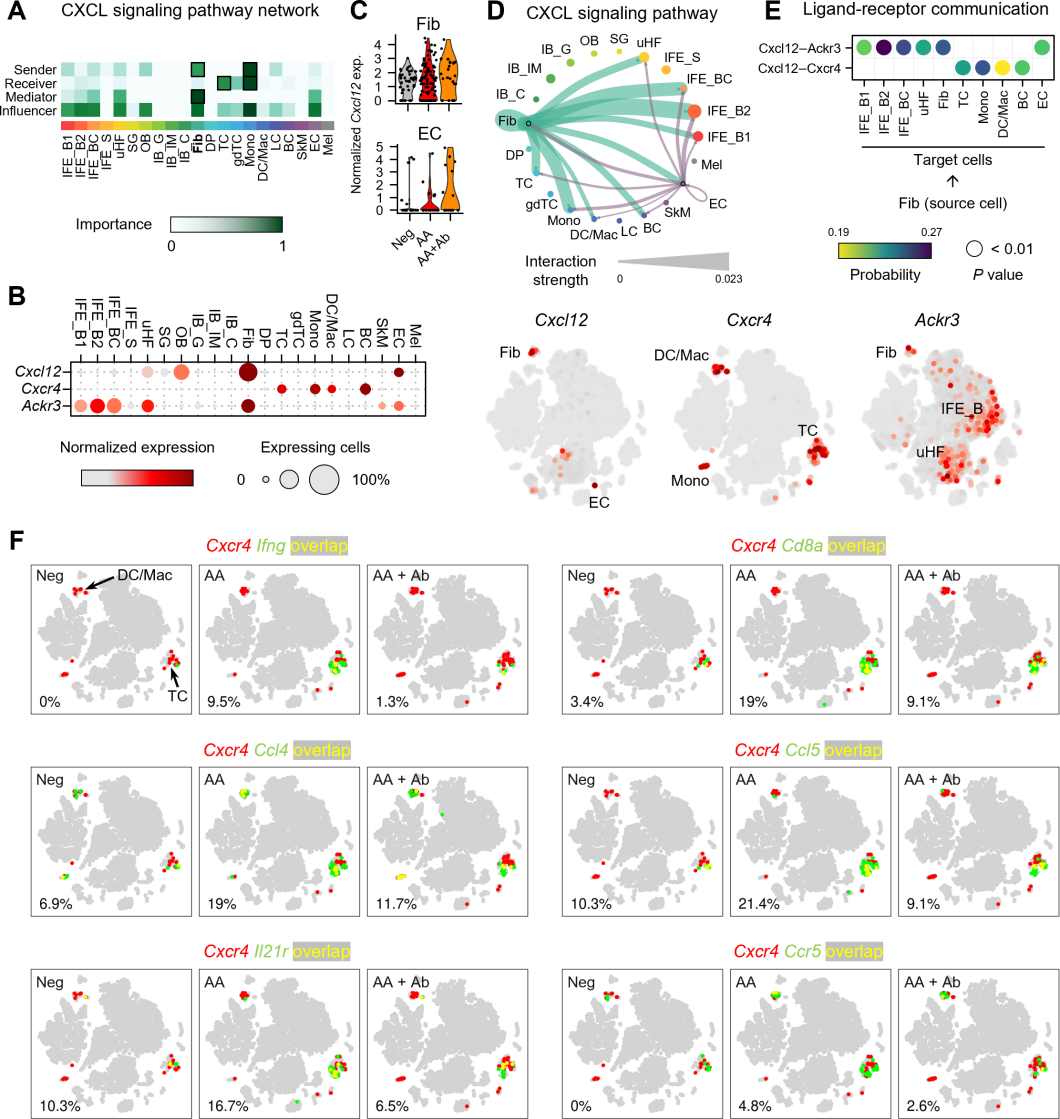
Expression profile of CXCL12 and its receptors in the AA mouse model. **(A)** Heatmap showing the relative importance of each cell type based on network centrality measures calculated from the CXCL signaling network using CellChat. **(B)** Expression levels of *Cxcl12* and genes of its receptors *Cxcr4* and *Ackr3*. **(C)** Normalized expression of *Cxcl12* in fibroblasts and ECs of each group. **(D)** Interaction strength between various cell types within the CXCL signaling pathway. The thickness of the lines connecting cell types is proportional to the calculated interaction strength. **(E)** Significant ligand-receptor pairs contributing to *Cxcl12* signaling from fibroblasts. Dot color and size represent calculated communication probability and *P* values from a one-sided permutation test. **(F)** Coexpression patterns of *Cxcr4* with DEGs related to immune cell activation in each group. In the t-SNE representation, cells expressing *Cxcr4* are shown in red, cells expressing *Ifng*, *Cd8a*, *Ccl4*, *Ccl5*, *Il21r*, or *Ccr5* are shown in green, and cells expressing both are shown in yellow. The percentage at the bottom left represents the proportion of cells expressing both *Cxcr4* and the selected genes among *Cxcr4*-expressing cells.

To investigate the mechanisms underlying the immune response in AA, we performed cell-cell communication analysis (**Figure 3**; **Supplementary Figure S6**). This analysis, based on ligand and receptor gene expression across all skin cells, revealed that ligands from fibroblasts and DP cells predominantly drive overall transcriptional changes (**Supplementary Figures S6A, B**). Particularly, CXCL signaling showed fibroblasts as the major source (sender), with T cells and monocytes as the major target cells (receiver) (**Figure 3A**; **Supplementary Figure S6C**). Specifically, *Cxcl12* was predominantly produced by fibroblasts, with EC being the second most abundant producers. (**Figure 3B**). Notably, the expression level of the Cxcl12 gene in fibroblasts and EC showed upregulation in the AA group, though it was not statistically significant (**Figure 3C**). Cell-cell interaction analysis indicated that fibroblast-derived CXCL12 is the dominant source driving CXCL12-mediated signaling in the skin (**Figure 3D**). Analysis of fibroblast subpopulations indicated that *Cxcl12* is primarily expressed in *Igfbp7*+ type B or *Sfrp1*+ type C fibroblasts (**Supplementary Figures S7A, B**) (27). In addition, coexpression analysis revealed an increased proportion of *Pdgfra*+ *Cxcl12*+ cells in the AA model (9.5%) compared to Neg (2.9%) and AA + Ab (4.2%) (**Supplementary Figure S7C**). We also examined the expression levels of canonical receptors for Cxcl12, Cxcr4 and Cxcr7 (*Ackr3* gene) (28). *Cxcr4* was primarily expressed in immune cells such as T cells, monocytes, and DC/Mac (**Supplementary Figure S8**), while *Ackr3* was expressed in keratinocytes (IFE_B and uHF) and fibroblasts (**Figure 3B**). Cell-cell interaction analysis showed that fibroblast-derived CXCL12 likely influencing immune cells more via CXCR4 than CXCR7 (**Figure 3E**).

To confirm the potential involvement of Cxcr4-mediated immune response identified in functional enrichment analysis, we next analyzed the coexpression of Cxcr4 and the selected DEGs among 153 DEGs (**Figure 3F**). Notably, the proportion of cells coexpressing *Cxcr4* and *Ifng* among *Cxcr4*-expressing cells was 0%, 9.5%, and 1.3% in Neg, AA, and AA + Ab groups, respectively, suggesting that the type II interferon-mediated immune response identified in the functional enrichment analysis may be mediated by Cxcr4 (**Figure 3E**). Additionally, *Cd8a* gene encoding cell surface glycoprotein found on most cytotoxic T cells, and genes of chemokines like Ccl4 and Ccl5, which are involved in recruiting these cells via Ccr5 (29), demonstrated similar coexpression patterns with *Ifng*. The coexpression of *Cxcr4* with *Il21r*, encoding interleukin-21 receptor that transduces the IL-21-mediated growth promoting the proliferation and differentiation of T cell, further supports the involvement of *Cxcr4* in orchestrating the immune landscape in AA (**Figure 3E**).

### Trajectory analysis revealed CD8+ T cell as a dominant population in AA

**Figure 4:**
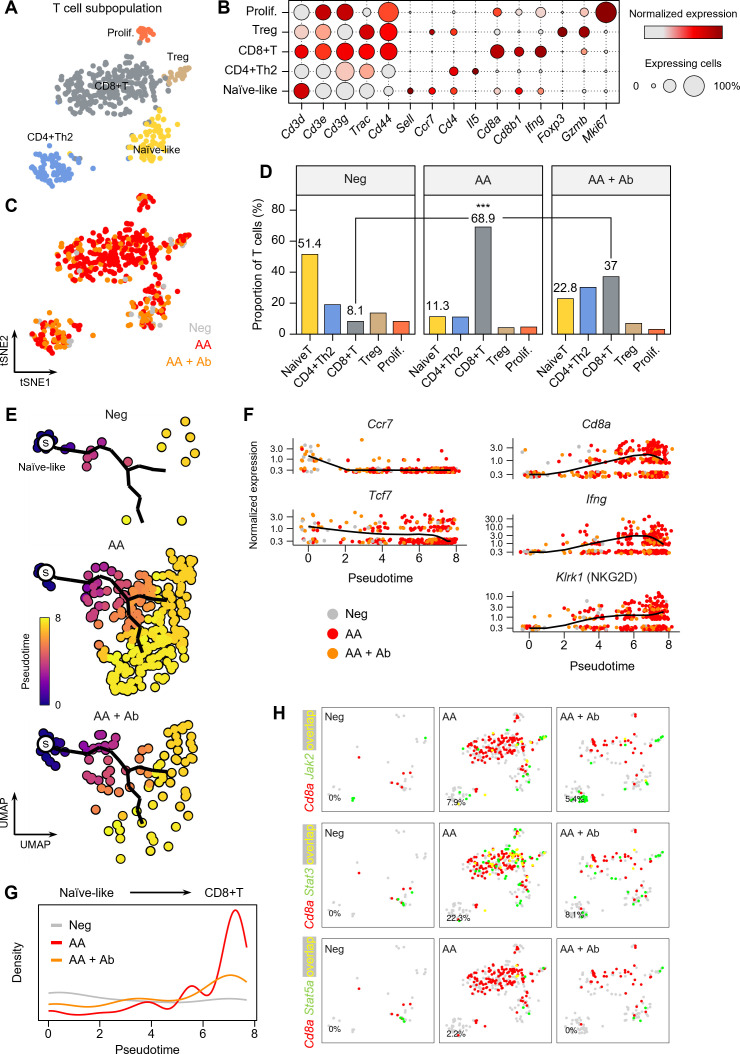
T cell subpopulations in the AA mouse model. **(A)** t-SNE representation of T cell subpopulations colored by detailed cell types. **(B)** Expression levels for selected marker genes of each cell type. **(C)** t-SNE representation of T cells colored by group (Neg, AA, or AA + Ab). **(D)** Proportion of each cell type among total T cells. Statistical significance was assessed using the binomial test. *** *P* < 0.001. **(E)** Pseudotime analysis of naïve-like T cells and CD8+ T cells. ‘s’ indicates the designated starting point (root) on the trajectory constructed based on UMAP representation. **(F)** Expression levels of selected marker genes over pseudotime. **(G)** Density of cells at each pseudotime by group. **(H)** Coexpression patterns of *Cd8a* with Jak/Stat-related genes in each group. In the t-SNE representation, cells expressing *Cd8a* are shown in red, cells expressing *Jak2*, *Stat3*, or *Stat5a* are shown in green, and cells expressing both are shown in yellow. The percentage at the bottom left represents the proportion of cells expressing both *Cd8a* and the selected genes among *Cd8a*-expressing cells.

The T cell population more than doubled in the AA model (**Figure 1**), and the major DEGs included *Ifng* and the cytotoxic T cell marker *Cd8a* (**Figures 2**, **3**). Thus, we next examined the composition changes in T cell subpopulations in more detail. Based on the expression of signature genes, we identified five T cell subpopulations: naïve-like T cells (*Sell* and *Ccr7*), CD4+ Th2 cells (*Cd4* and *Il5*), CD8+ T cells (*Cd8a*, *Cd8b1*, and *Ifng*), regulatory T (Treg) cells (*Foxp3* and *Gzmb*), and proliferating T cells (*Mki67*) (**Figures 4A, B**). A 2D t-SNE plot marked by the groups (Neg, AA or AA + Ab) qualitatively showed an increase in CD8+ T cells in the AA model (**Figure 4C**). Quantitative cell composition analysis revealed that the proportion of CD8+ T cells in the total T cell population significantly increased from 8.1% in Neg to 68.9% in AA, then decreased to 37% with Ab treatment (**Figure 4D**).

We further performed pseudotime analysis to investigate peripheral activation and maturation process of T cells in AA, focusing on naïve-like T cells and CD8+ T cell populations. Using naïve-like T cells as the starting point, we traced the trajectory of cells activating into CD8+ T cells (**Figure 4E**). Early naïve-like T cells predominantly expressed markers of resting T cells such as *Ccr7* and *Tcf7* (30), while along the pseudotime trajectory, T cells showed increased expression of *Cd8a* and *Ifng* (**Figure 4F**). Terminally activated T cells exhibited characteristics of CD8+ NKG2D+ effector T cells, which play a crucial role in AA induction (31). Notably, in the AA model, we observed a significant increase in the distribution of terminally activated CD8+ T cells corresponding to pseudotime 6 to 8, while their proportion decreased with CXCL12 Ab treatment (**Figure 4G**). Coexpression analysis of signaling pathways contributing to T cell activation revealed a high overlap between Cd8a and Jak2, Stat3, and Stat5a in the AA model, suggesting the involvement of these pathways in AA pathogenesis (**Figure 4H**).

## Discussion

We investigated the underlying molecular mechanisms of humanized CXCL12 Ab in the treatment of AA. Abdominal skin of AA mice was analyzed using scRNA-seq. T cells and dendritic cells/macrophages were increased in the AA model and decreased after CXCL12 Ab treatment. Notably, CD8+ T cells significantly increased in the AA model and decreased after CXCL12 Ab treatment. Pseudobulk RNA sequencing identified 153 DEGs that were upregulated in AA model and downregulated after Ab treatment. GO analysis revealed that immune cell chemotaxis and cellular response to type II interferon were upregulated in AA mice and downregulated after Ab treatment. We further identified key immune cell-related genes such as *Ifng, Cd8a, Ccr5, Ccl4, Ccl5*, and *Il21r* regulated by CXCL12 Ab treatment. Collectively, these results indicate that humanized CXCL12 Ab is effective for AA treatment via immune modulatory effects.

AA is due to the loss of immune privilege of the hair follicle, leading to autoimmune attack, and many studies have reported the key immune cells responsible for hair follicle damage (32). Although the etiopathogenesis of AA has not yet been fully characterized, the collapse of immune privilege at the hair follicle followed by T cell receptor recognition of exposed hair follicle autoantigens by autoreactive cytotoxic CD8+ T cells is now understood to play a central role (32, 33). Recently, functional interrogation of lymphocyte subsets in AA was analyzed in mice and humans using scRNA-seq, and it was found that CD8+ T cells are the predominant disease-driving cell type (10). Although CD8+ T cells are the predominant expanded cell type in mouse AA skin, CD4+ Treg cells, NK T cells, and γδ T cells are also increased in human AA skin (34). In addition, functional roles for CD4+ T cells and antigen-presenting cells have been suggested in AA using single-cell analysis (11). In this study, AA onset in mice was induced by lymph node cell injection, resulting in significant increases in CD8+ T cells and dendritic cells/macrophages, which led to hair loss. Conversely, subcutaneous injection of CXCL12 Ab significantly reduced these immune cells in the skin, delaying AA onset.

DFs are an important subset of mesenchymal cells in maintaining skin homeostasis and resisting harmful stimuli. DFs modulate immune cell function by secreting cytokines, thereby implicating their involvement in various dermatological conditions such as psoriasis, vitiligo, and atopic dermatitis (35). Recently, histological approaches and scRNA-seq studies on human skin diseases have revealed fibroblast subsets with unexpected immuno-modulatory transcriptomes and immune cell changes, suggesting a potential role for these cells in the pathogenesis of inflammatory skin disorders (36). DFs play a pivotal role as a cellular source of inflammatory cytokines and chemokines, promoting chronic tissue inflammation through leukocyte recruitment and exacerbating inflammatory injury (37). Although we did not further investigate other cytokines and chemokines, CXCL12 is highly expressed and secreted from DFs, and mediates inflammatory and immune stimulatory milieu to induce AA.

CXCL12 receptors (i.e., CXCR4 and CXCR7) are belonging to the G protein-coupled receptors family, and are abundantly expressed in diverse skin and hair cells (**Figure 3C**). CXCR7 also bind to CXCL11 and is highly expressed in DFs and keratinocytes, but its function has not been fully demonstrated. However, pharmacological effects of CXCR4 are well-known in skin and hair. For example, CXCR4 is expressed in keratinocytes, and mediates wound-healing effects. It is also expressed in ORS cells and regulates hair cycle. However, single cell analysis revealed that CXCR4 is highly expressed on immune cells such as T cells, DC, and macrophage, and is co-expressed with inflammatory cytokines and chemokines (**Figure 3**). On that account, it is reasonable to assume that CXCL12 secreted from DFs activates immune cells via CXCR4 pathway. Therefore, the upregulation of inflammatory factors such as IFNG and CCL families in T cells increases the proliferation and migration of T cells to attack hair follicles. Consequently, CXCL12 Ab attenuates hair loss in AA by inhibiting T cell activation through CXCR4 signaling pathway.

Hair loss can result from various factors, and current treatments include topical medications and oral drugs. In the case of AA, topical application of Jak inhibitors is not as effective as oral administration because AA is not restricted to the hair follicle and is regulated by the immune system (38, 39). Jak inhibitors are efficacious and generally well-tolerated for treating AA with oral administration (40). However, due to the high recurrence rate after withdrawal of Jak inhibitors, continuous treatment is necessary to maintain efficacy. Since Jak inhibitors are administered daily, developing a long-lasting solution is crucial to enhance the effectiveness and convenience of AA treatments. Notably, Ab therapy has many advantages due to its long-lasting effects in AA treatment. Because the molecular weight of Ab is very high, it is difficult to penetrate the capillary in the scalp and it is slowly absorbed through the lymphatic system (41, 42). Therefore, many subcutaneous antibody medications are administered at regular intervals, often monthly or bimonthly. Additionally, direct injection of antibody medications into the hair loss areas is possible, leading to superior treatment efficacy in AA. Due to the long duration of action and absorption via the lymphatic system, CXCL12 Ab therapy is highly promising for AA treatment.

## Data Availability

The original data generated and analyzed in this study are publicly available in the NCBI Gene Expression Omnibus (GEO) repository under the accession number GSE269455. The datasets can be accessed at: https://www.ncbi.nlm.nih.gov/geo/query/acc.cgi?acc=GSE269455.
