# Supplementary material for: Humanized CXCL12 antibody delays onset and modulates immune response in alopecia areata mice: insights from single-cell RNA sequencing
Source: Front Immunol. 2024 Oct 17;15:1444777. doi: 10.3389/fimmu.2024.1444777 (PMC11524852; doi:10.3389/fimmu.2024.1444777)
Supplement: Supplementary file 1 [file DataSheet1.pdf]

Supplementary Table S1. Summary of cell type annotations

	Short name	Cell type	Selected marker gene	# of cells
Epidermis keratinocytes	IFE B	Interfollicular epidermis, basal	*Krt5, Krt14*	9,118
	IFE BC	Interfollicular epidermis, basal (cycling)	*Krt5, Krt14, Stmn1, Mki67*	339
	IFE S	Interfollicular epidermis, suprabasal	*Krt1, Krt10*	2,437
	uHF	Upper hair follicle	*Krt17, Krt79*	3,556
	SG	Sebaceous gland	*Scd1, Mgst1*	128
	OB	Outer bulge	*Barx2*	512
Hair follicle keratinocytes	IB G	Germinative layer	*Krt27, Krt35*	1,896
	IB IM	Inner root sheath & medulla	*Krt27, Krt35*	2,549
	IB C	Cortex / cuticle	*Krt27, Krt35*	309
Fibroblast-like cells	Fib	Fibroblast	*Col1a1, Lum*	178
	DP	Dermal papilla	*Corin, Notum*	176
Immune cells	(gd)TC	($\gamma\delta$)T cell	*Cd3e, Trdc*	1,137
	Mono	Monocyte	*Cd14, Ccl6*	136
	DC/Mac	Dendritic cell & macrophage	*Cd68, Cd74*	223
	LC	Langerhans cell	*Cd207*	40
	BC	B cell	*Cd79a*	43
Others	SkM	Skeletal muscle	*Acta1, Des*	266
	EC	Endothelial cells	*Pecam1, Cdh5*	55
	Mel	Melanocyte	*Pmel, Dct*	124

Supplementary Table S2, 3

Attached EXCEL file

Supplementary Figure S1

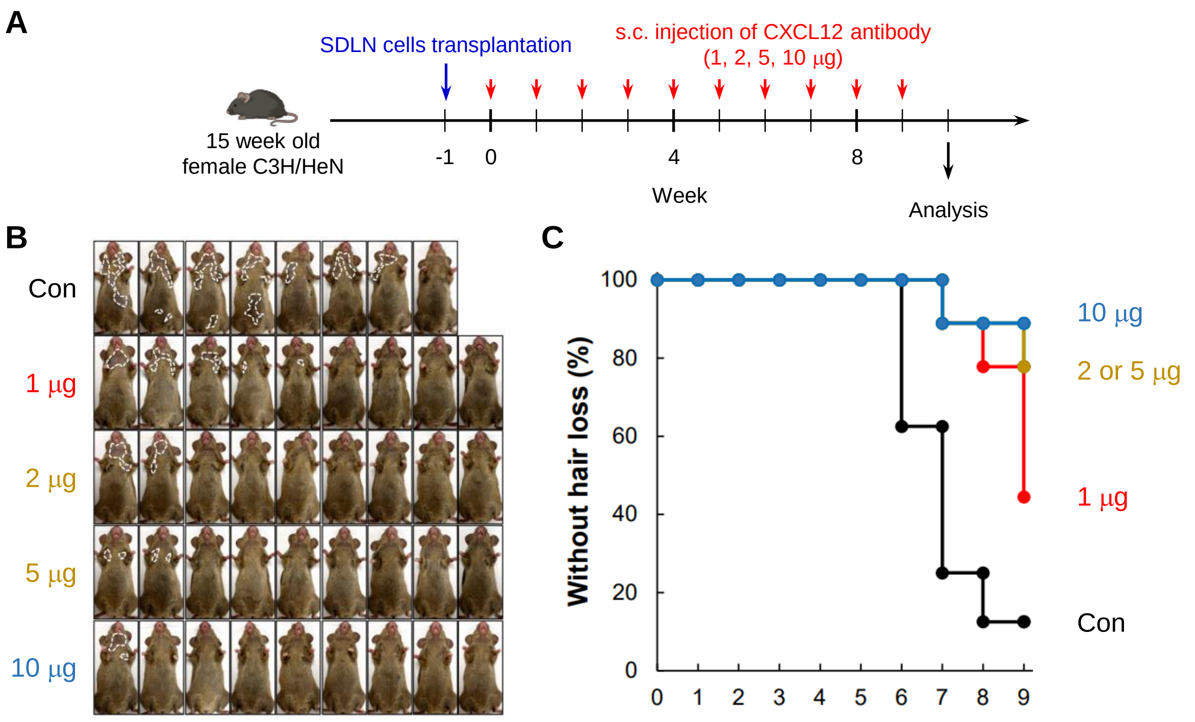

AA mouse model and treatment with CXCL12 Ab. (A) Skin-draining lymph node (SDLN) cells were isolated from AA-affected C3H/HeN female mice and injected intradermally into the dorsal skin of naïve mice to induce AA. The CXCL12-neutralizing Ab was administered subcutaneously once a week for ten weeks. (B) The regions of skin induced with AA were manually delineated. (C) The proportion of animals without hair loss was recorded.

Supplementary Figure S2

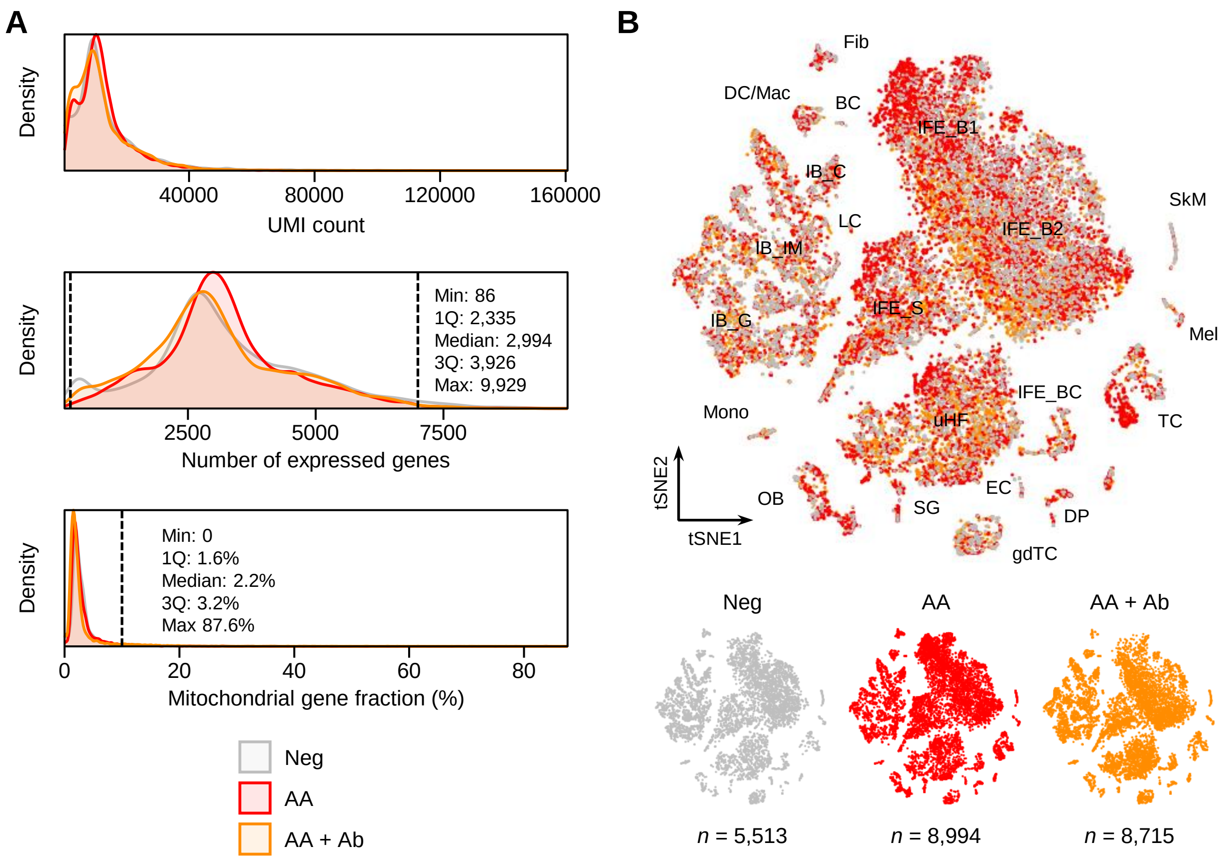

Quality control of scRNA-seq data. (A) Initial quality control filtering. Cells expressing between 200 and 7,000 genes, and with mitochondrial gene expression fraction below 10%, passed the initial quality control filter. The dashed lines on the density plot represent the filter criteria. (B) t-SNE representation of scRNA-seq results from Neg, AA, and AA + Ab groups, with each group represented by a different color. The number of cells in each group is also labeled.

Supplementary Figure S3

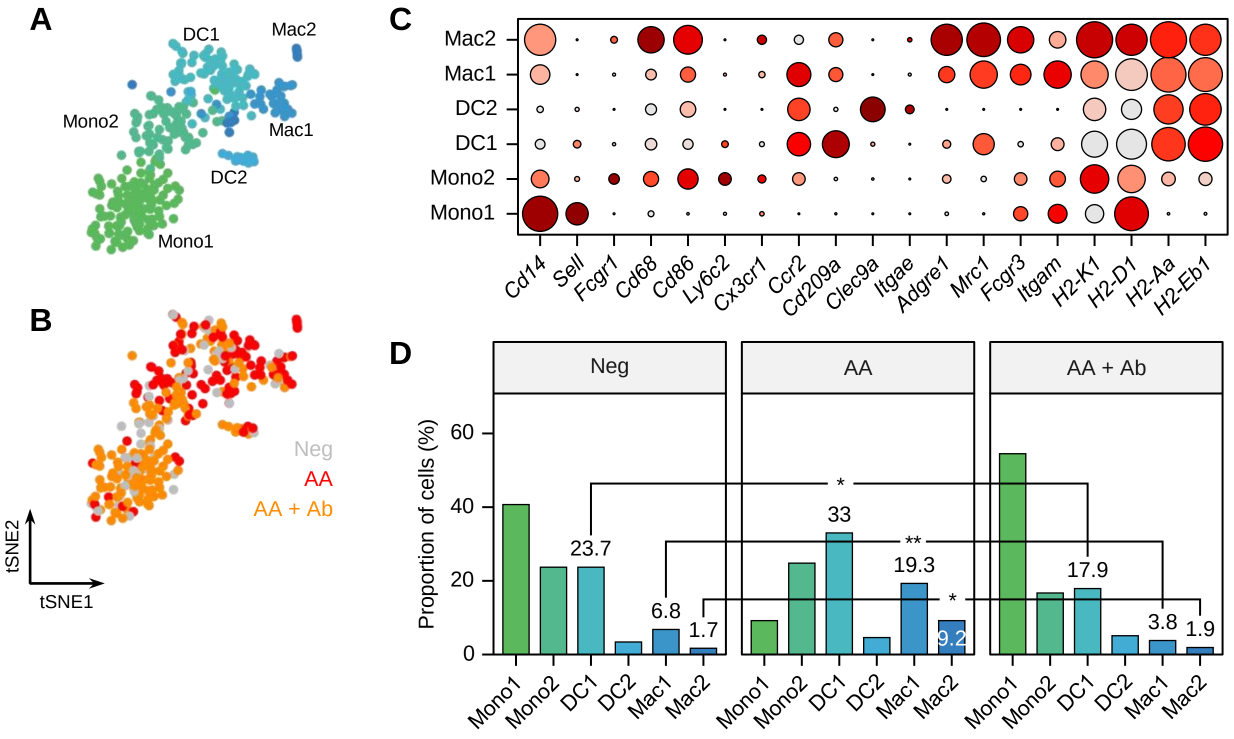

Monocytes and DC/Mac subpopulations. (A) t-SNE representation of monocytes and DC/Mac subpopulations colored by detailed cell types. (B) t-SNE representation colored by groups (Neg, AA, or AA + Ab). (C) Expression levels for selected marker genes of each cell type. (D) Proportion of each cell type among monocytes and DC/Mac subpopulations. Statistical significance was assessed using the binomial test. * $P < 0.05$ and ** $P < 0.01$.

Supplementary Figure S4

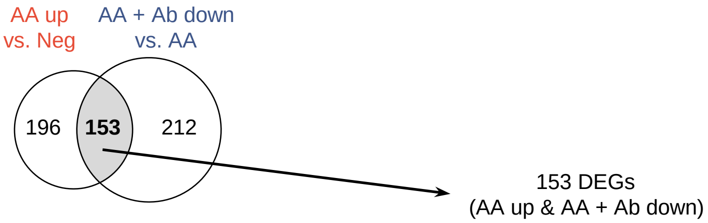

Abcg3	Ccdc80	Cd8a	Cyp26b1	Gbp2b	Igflr1	Ly86	Pdcd1	Sema6d	Tead4	Zbp1
AI504432	Ccer2	Cd8b1	Cyp2f2	Gbp5	Igtp	Lyz1	Pde4a	Serpinf1	Tgm5	Zfp385b
Angptl7	Cck	Ces1d	Dchs2	Gbp6	Iigp1	Mrc1	Penk	Serping1	Tmem117	Zfp521
Apod	Ccl11	Cfb	Dnm3os	Gbp8	Ikzf3	Ms4a4b	Pf4	Sgcd	Tmem119	
Apol9a	Ccl4	Cgref1	Dusp4	Gbp9	Il21r	Nalcn	Phlda1	Slamf1	Tmem132c	
Apol9b	Ccl5	Chat	Dusp6	Gfpt2	Inmt	Ncam2	Pld5	Slamf7	Tnfrsf11b	
Bcl2a1d	Ccl8	Chrdl1	Egflam	Gpr174	Irgm1	Nfatc4	Ptgfr	Slc26a7	Tnfsf14	
C1qa	Ccl9	Cilp	Erbb4	Gpr55	Irgm2	Ngf	Pyy	Slc2a13	Tnnt1	
C1qb	Ccr5	Clec10a	F13a1	Gzmb	Itga1	Npr3	Retnla	Sobp	Tppp3	
C1qc	Cd101	Clmp	Fam13a	Icos	Kcnd3	Oas1a	Rfx2	Sorcs3	Trgv2	
C1qtnf3	Cd209a	Col1a1	Fasl	Ifi205	Klrc1	Olfml1	Rtp4	Sparcl1	Tsc22d2	
C1rb	Cd209d	Col1a2	Fcrls	Ifi211	Krt16	Olfml2b	Runx1t1	Srpx	Tspan11	
C1s1	Cd226	Cubn	Fosl1	Ifi44	Lepr	Olfml3	Rxfp1	Srpx2	Ubd	
C1s2	Cd40	Cxcl9	Fut9	Ifi47	Lox	Olfr60	Scara3	Tacr1	Wdr66	
C4b	Cd6	Cxcr3	Gbp2	Ifng	Lvrn	Pamr1	Scara5	Tbx21	Wfdc18	

List of DEGs related to AA treatment with CXCL12 Ab. A total of 153 intersecting genes were identified that showed over a twofold increase in expression in the AA group compared to the Neg group, and over a twofold decrease in expression in the AA + Ab group compared to the AA group.

Supplementary Figure S5

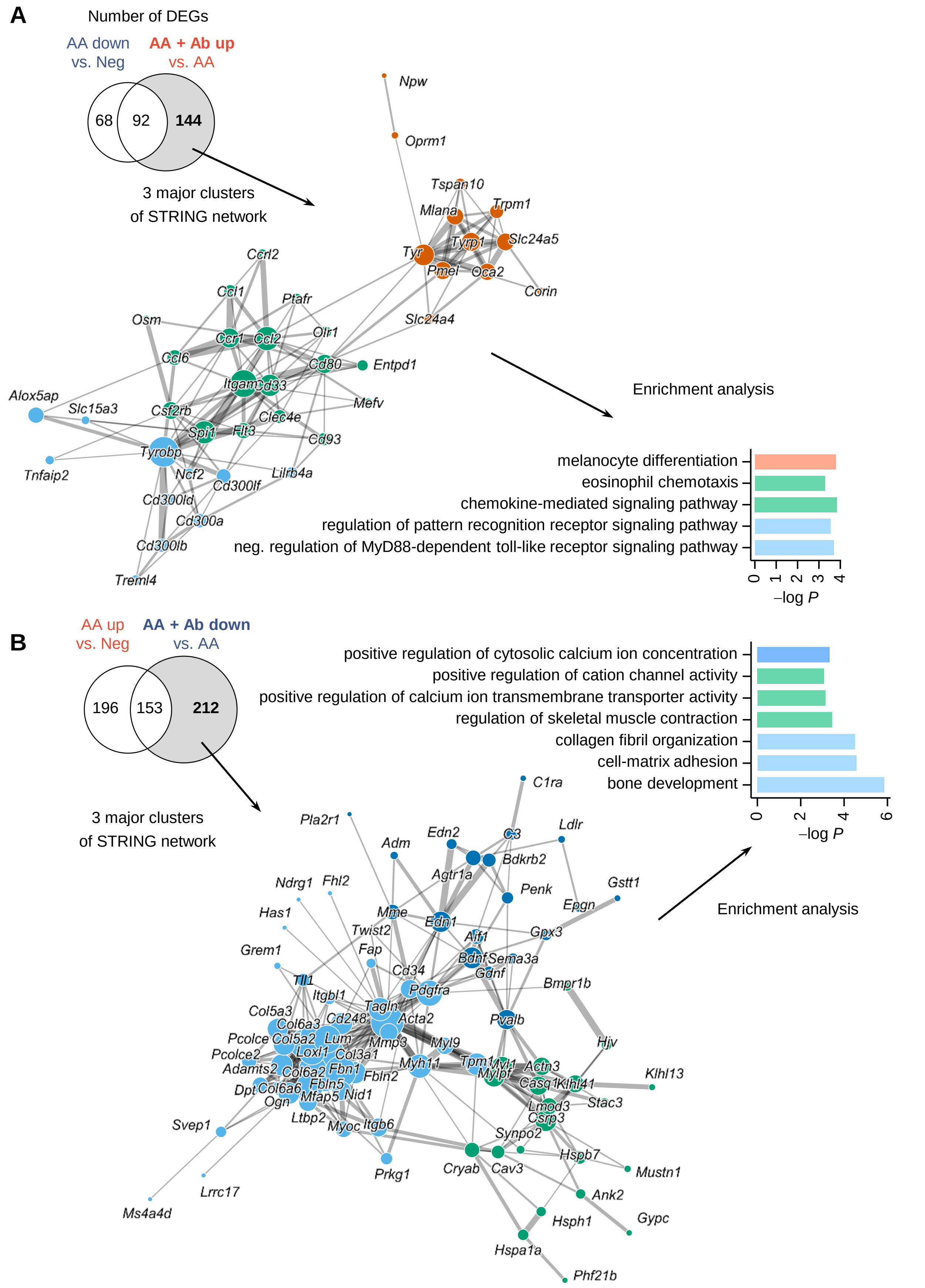

CXCL12 Ab-specific pseudobulk transcriptomic changes. DEGs were identified based on a more than twofold change in expression in comparisons of Neg vs. AA and AA vs. AA + Ab (Figure 3). Ab-specific upregulated genes (A) and downregulated genes (B) were analyzed separately. STRING networks were constructed to visualize protein-protein interactions for each gene set. The three main clusters identified through community detection, using weighted edge betweenness, were subjected to GO enrichment analysis. Significant results with a *P* value < 0.001 are shown.

Supplementary Figure S5 (continued.)

C

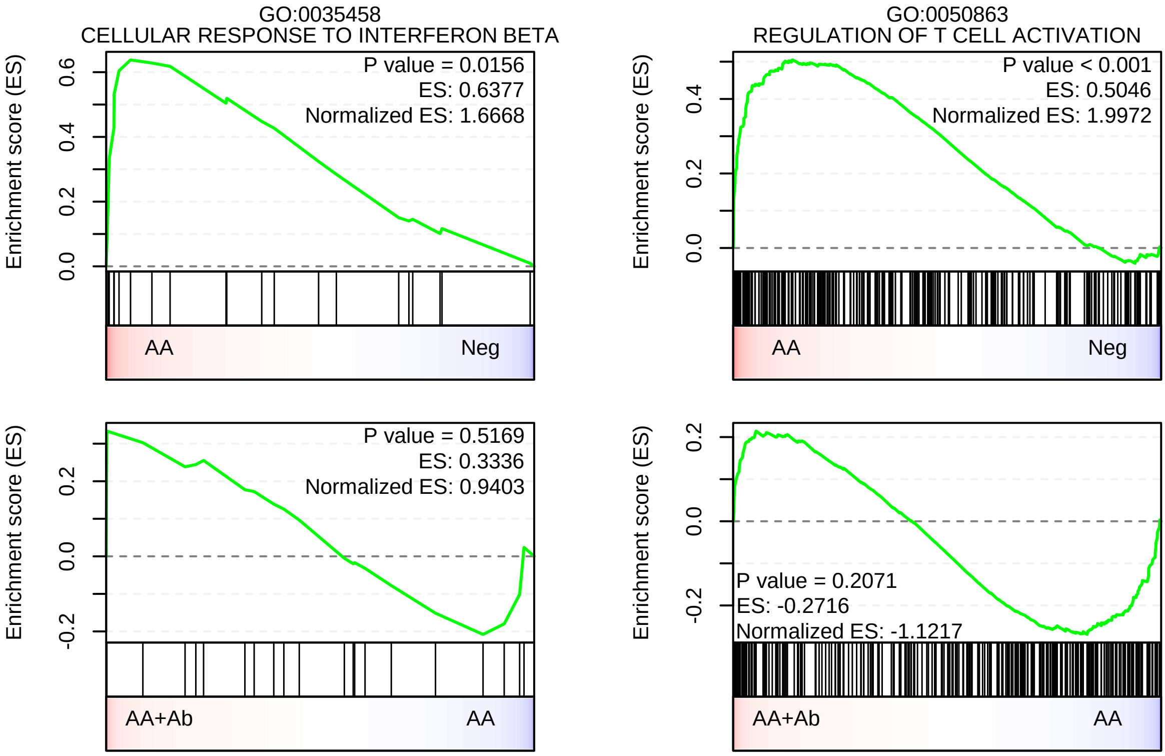

(C) GSEA results for aggregated pseudobulk RNA-seq data. Gene Set Enrichment Analysis (GSEA) was performed using $\log_2$ fold change values calculated from comparisons of AA vs. Neg and AA + Ab vs. AA. The pre-ranked GSEA utilized these $\log_2$ fold change values as input.

Supplementary Figure S6

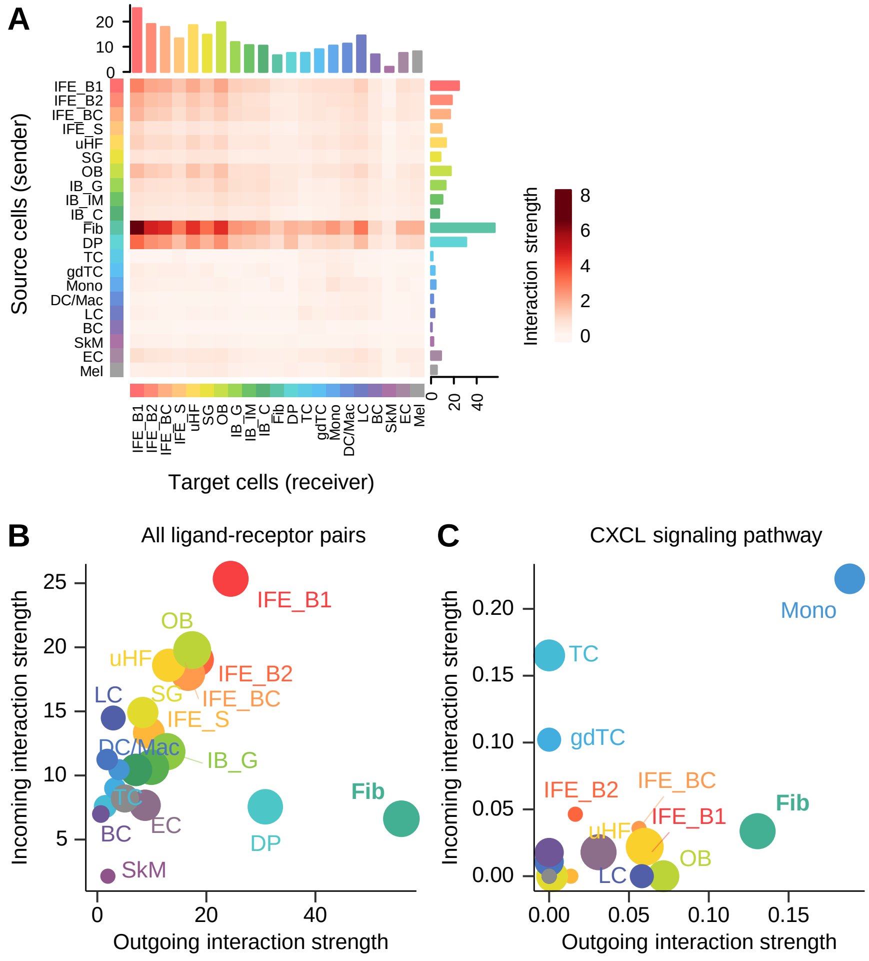

Overview of cell-cell communication analysis. (A) Using CellChat, interaction strength was calculated for 3,373 ligand-receptor pairs between cell types. Interaction strength is determined by the expression level of the ligand gene in the source cell (sender) and the receptor gene in the target cell (receiver). (B) For each cell type, incoming and outgoing interaction strengths were calculated for all ligand-receptor pairs. Dot size is proportional to the number of significant interactions involved. (C) For the CXCL signaling pathway, incoming and outgoing interaction strengths were calculated for each cell type based on the relevant ligand-receptor pairs.

Supplementary Figure S7

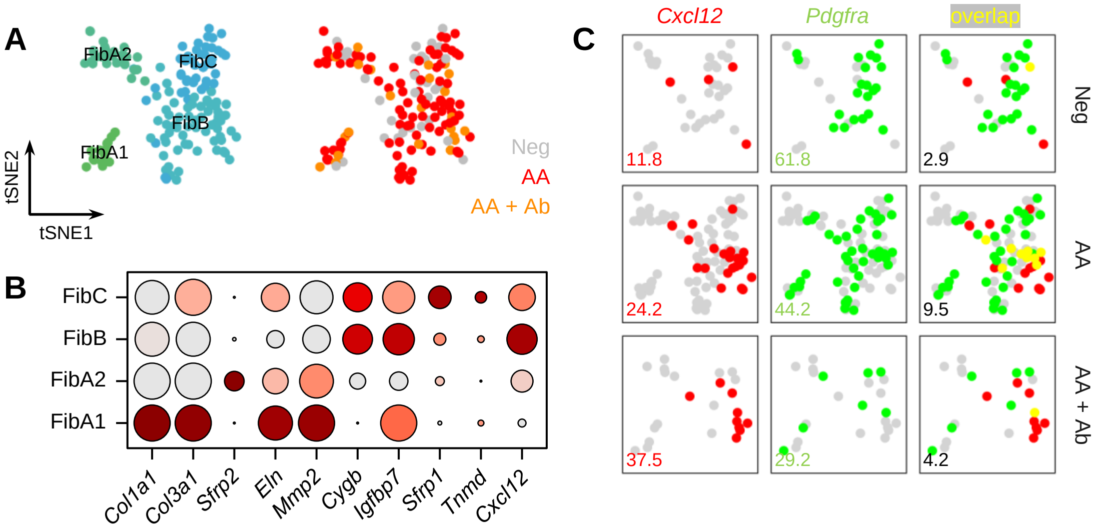

Fibroblast subpopulations. (A) t-SNE representation of derma fibroblast subpopulations colored by detailed cell types (left) or groups (right: Neg, AA, or AA + Ab). The subpopulations are identified as FibA (fibroblast type A), FibB (fibroblast type B), and FibC (fibroblast type C). (B) Expression levels for selected marker genes of each cell type. (C) Coexpression patterns of *Cxcl12* and *Pdgfra* in each group. In the t-SNE representation, cells expressing *Cxcl12* are shown in red, cells expressing *Pdgfra* are shown in green, and cells expressing both are shown in yellow. The percentage at the bottom left represents the proportion of cells expressing both *Cxcl12* and *Pdgfra* among *Pdgfra*-expressing cells.

Supplementary Figure S8

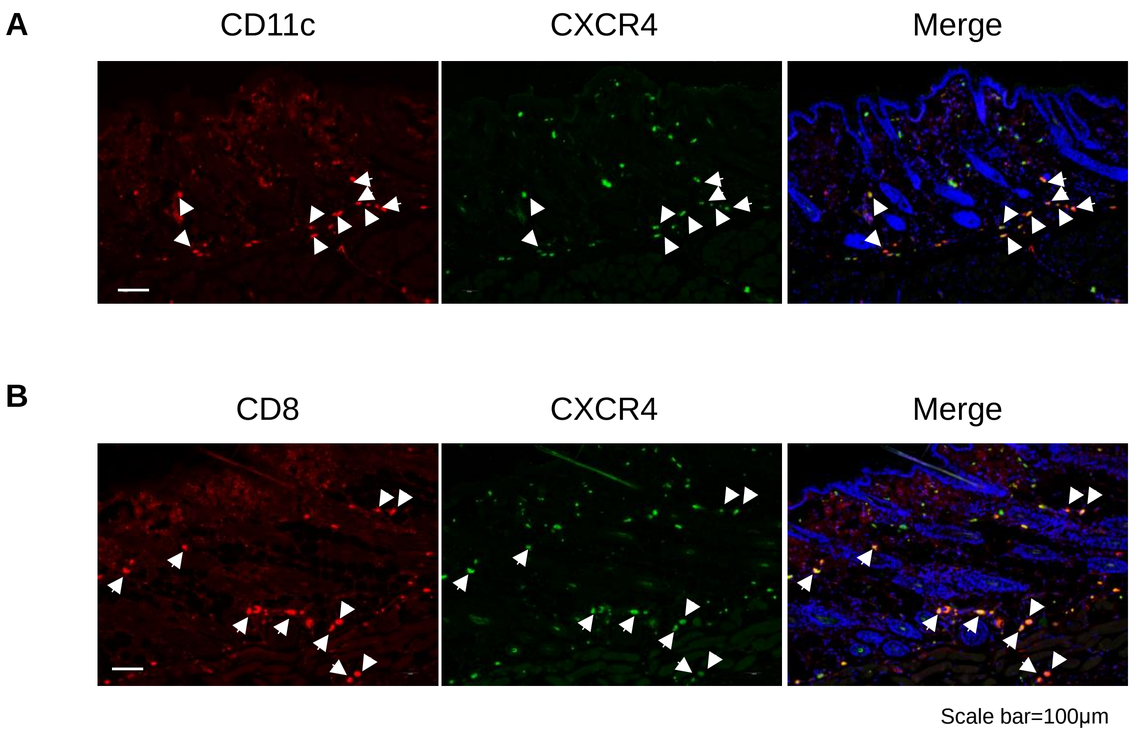

Coexpression of CXCR4 and immune cell markers. Skin sections of AA mice were stained with anti-CXCR4 together with anti-CD11c (A) or anti-CD8 (B). Scale bar=100 μm.
